# The lived experiences of women with polycystic ovary syndrome and its psychological challenges: A systematic review and meta-synthesis

**DOI:** 10.1007/s00737-025-01636-4

**Published:** 2026-02-12

**Authors:** Faathimah Khan, Nalini Govender, Sara Bibi Mitha, Yasmeen Thandar

**Affiliations:** 1 Dept of Basic Medical Sciences, Durban University of Technology, Durban, South Africa; 2 Alan Pittendrigh Library, Steve Bhiko Campus, Durban University of Technology, Durban, South Africa

**Keywords:** Polycystic ovary syndrome, Mental health, Psychosocial factors, Women's health, Qualitative research

## Abstract

**Purpose:**

Polycystic ovary syndrome (PCOS) presents psychological challenges in women due to persistent symptoms such as hirsutism, irregular menstruation, and weight gain, and long-term complications like infertility. Women with PCOS face higher risks of depression, anxiety, body image dissatisfaction, and social challenges. Research often overlooks the psychological burden of PCOS, despite its documented effects. This systematic review aims to explore the lived psychological experiences of women with PCOS, encompassing mental health challenges, emotional well-being, and psychosocial factors. It also aims to identify sociocultural variations in psychosocial challenges.

**Methods:**

A systematic search was conducted across six bibliographic databases (MEDLINE, Web of Science, Cochrane, CINAHL, PubMed, and SCOPUS) from inception to April 2024. Qualitative and mixed-methods studies in English exploring the psychological experiences of women with PCOS were included. Study quality was assessed using the Joanna Briggs Institute Qualitative Assessment and Review Instrument (JBI-QARI), and findings were synthesized using a JBI-guided pragmatic meta-aggregation approach.

**Results:**

A total of 43 studies were included, yielding 240 unequivocal findings and 46 credible findings which were meta-aggregated into two themes: (1) Mental Health Challenges associated with PCOS (2) Psychosocial Challenges associated with PCOS.

**Conclusion:**

Depression, anxiety, body image dissatisfaction, and social stigma significantly impact women with PCOS. Hirsutism and being overweight are prominent stressors, particularly affecting psychological well-being in this group. Sociocultural factors play a role in shaping psychological experiences. Effective PCOS care requires mental health support, fertility counselling, and culturally sensitive interventions to improve health outcomes.

**Supplementary Information:**

The online version contains supplementary material available at 10.1007/s00737-025-01636-4.

## Highlights

PCOS symptoms (e.g., hirsutism, weight gain, irregular menses, infertility) are key biological stressors that trigger psychological distress.

The meaning and intensity of this distress (e.g., depression, anxiety, low self-worth) are shaped by sociocultural expectations of femininity, appearance, and motherhood.

Infertility and visible symptoms intensify stigma, social comparison, and relationship strain, reinforcing feelings of inadequacy and isolation.

Psychological challenges evolve across the life course, intersecting with changing cultural and social pressures.

The Biopsychosocial Model illustrates how PCOS is not only a medical condition but also a psychosocial and existential burden, underscoring the need for culturally sensitive, holistic interventions.

**Supplementary Information:**

The online version contains supplementary material available at 10.1007/s00737-025-01636-4.

## Introduction

Polycystic ovary syndrome (PCOS), affecting 4–20% of women globally, is a complex reproductive endocrinopathy with clinical and psychological impacts (Deswal et al. 2020). Characterized by irregular menstruation, hyperandrogenism and polycystic ovaries, PCOS manifests with symptoms like acne, weight gain, and hirsutism (Teede et al. 2010). PCOS also increases long-term risks of type 2 diabetes, infertility, and cardiovascular disease (Joham et al. 2022). These clinical implications significantly affect mental health (Farajzadegan et al. 2023), treatment adherence and quality of life (QoL), thus warranting urgent investigations to understand and disrupt this cycle (Hu et al. 2024). However, most PCOS research often prioritizes clinical aspects over psychological ones (Teede et al. 2010).

Mental health challenges remain underexplored despite PCOS increasing susceptibility to depression, anxiety, eating disorders, and body image distress (Hu et al. 2024). Although mental well-being is often overlooked in clinical care (Farajzadegan et al. 2023), systematic reviews such as those by Alur-Gupta & Dokras (2022) and Rodrigues-Paris and colleagues (2019) confirm heightened risks of psychiatric conditions and reduced QoL.

Multiple studies have shown strong associations between PCOS and anxiety disorders (Cooney et al. 2017; Dokras 2012; Hart & Doherty 2015). A meta-analysis by Dokras (2012) reported a higher prevalence of generalized anxiety disorder in women with PCOS, reinforced by Hart and Doherty (2015) (14.0% vs. 5.9%) and Cooney et al. (2017), who reported a six-fold risk. Depression is similarly prevalent, linked to serum androgens, infertility, and obesity (Cesta et al. 2017; Dokras 2012; Rodriguez-Paris et al. 2019). Studies by Cesta et al. (2017) and Cooney et al. (2017) corroborate this association, highlighting a higher risk of depression among women with PCOS. A meta-analysis by Cooney et al. (2017) further reported moderate and severe depression scores, with an odds ratio of 4.18 (95% CI: 2.68–6.52; 11 studies). Body image dissatisfaction is also commonly reported, as shown by Farajzadegan et al. (2023) and Morshedi et al. (2021), with many women experiencing feelings of unattractiveness and a perceived loss of femininity, leading to reduced self-esteem compared to women without PCOS. Eating disorders are also more prevalent in this population, as Lee et al. (2017) reported a 35% increased risk of bulimia. Anxiety often compounds these challenges, elevating disordered eating risks regardless of body mass (Rodriguez-Paris et al. 2019).

Women with PCOS face a range of psychosocial challenges that impact their QoL. Emotional reactions to these challenges include sadness, anger, stress (Podfigurna-Stopa et al. 2015; Sayyah-Melli et al. 2015), insecurity, and frustration (Wright et al. 2020), which plays a significant role in long-term mental health issues (Zhaoyang et al. 2020). These challenges often manifest as inadequacy, withdrawal, depressive symptoms, and heightened social fears that reduce social functioning and well-being (Moreira et al. 2010; Sayyah-Melli et al. 2015). PCOS also strains sexual and social functioning, with changes in appearance, mood, and fertility linked to reduced intimacy, lower self-esteem, and in severe cases, marital breakdown or diminished social status (Amiri et al. 2014a; Brady et al. 2009). Infertility remains a major cause of distress, affecting both women and their partners (Hadjiconstantinou et al. 2017; Zaikova 2021). On a broader level, appearance-related symptoms and fertility concerns fuel social anxiety and isolation, often intensified by societal expectations around femininity and motherhood (Farajzadegan et al. 2023; Morshedi et al. 2021). While these sociocultural influences are recognized, more research is needed in diverse cultural contexts to understand global variation (Alur-Gupta et al. 2021; Bhatti et al. 2024; Borghi et al. 2018; Cinar et al. 2011; Elsenbruch et al. 2003) (see Supplementary Information SS1).

Thus far, clinical research has often overlooked the psychological impacts of PCOS from women’s perspectives. This qualitative systematic review (SR) thus aims to:

1. Explore the psychological dimensions of PCOS, including mental health, emotional well-being, and psychosocial aspects, within the context of the Biopsychosocial Model.
2. Document potential variation in psychosocial experiences among different sub-populations of women with PCOS.

Guided by the research question: "*What are the psychological challenges (including mental health, emotional, and psychosocial aspects) experienced by women with PCOS?”*, this SR applies the Biopsychosocial Model (Engel 1980) as a comprehensive lens. The model emphasizes how biological manifestations of PCOS interact with psychological responses and sociocultural expectations, making it particularly suited to capture the complexity of women’s lived experiences.

## Methods

### Research design

This SR was performed using the Joanna Briggs Institute (JBI) meta-synthesis approach (Aromataris et al. 2020) and was registered on PROSPERO on April 30th, 2024 [ID: CRD42024540166].

### Search strategy

The following databases were searched: MEDLINE, Web of Science, Cochrane, CINAHL, PubMed and SCOPUS. To ensure a comprehensive search, we used a three-step strategy. First, a preliminary PubMed search before PROSPERO registration assessed the review’s viability and informed the formal search design. Second, we applied a refined search formula with all relevant keywords and index terms across target databases. Third, we manually screened reference lists to identify any additional studies. Key terms included: “Polycystic ovary syndrome”, “polycystic ovar*”, “stein-Leventhal syndrome”, “PCO*”, “experience”, “first-hand”, “narrative”, “perspective”, “experiential knowledge”, “interview*”, “opinion*”, “qualitative”, “mixed methods”, “action research”, “feminist research”, “ground theory”, “phenomenology”, “psychological”, “emotional”, “mental”, “psychiatric disorder”, “mood disorder*”, “eating disorder*”, “sleeping disorder*”, “psychosocial*”, “psychosexual*”, “anxiety”, “depression”, “social anxiety”, “social isolation”, “self-esteem”, “self-image”, “body image”, “sexual functioning”, “sexual disorder”. The search term included medical subject headings and free words. Data were retrieved from inception of database up to April 2024. The full search strategies for each database are provided in Supplementary File SS2.

### Eligibility criteria and study selection

**Fig. 1:**
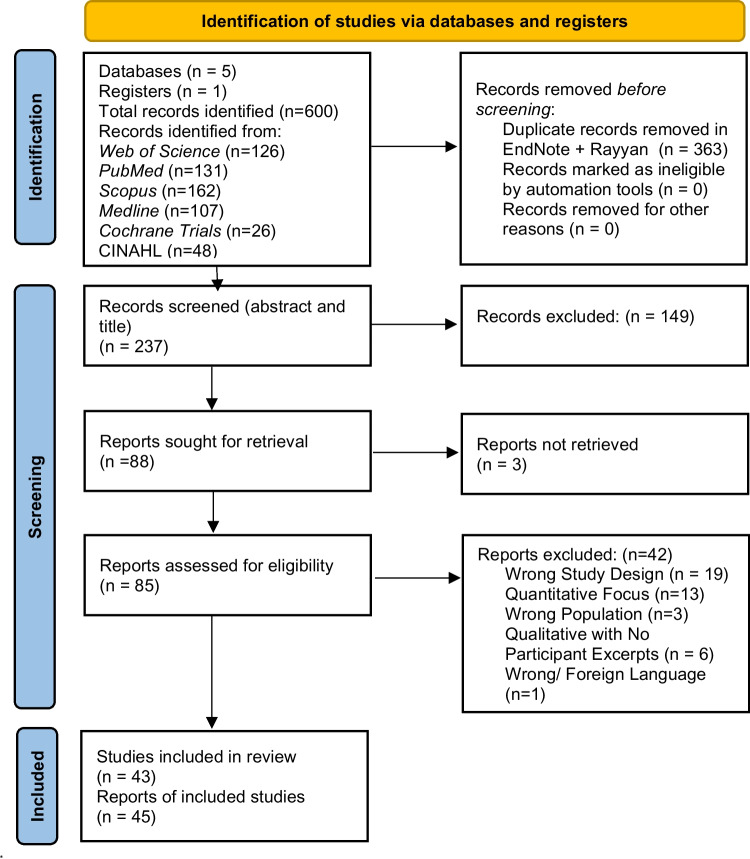
PRISMA flow diagram demonstrating search process

The inclusion and exclusion criteria of studies selected are shown in Table 1. The selection process is presented in the Preferred Reporting Items for Systematic Reviews and Meta-Analyses (PRISMA) flowchart (Fig. 1). Three researchers (F.K, Y.T, and N.G) independently screened all titles and abstracts, and disagreements in the screening process were subsequently resolved through discussion.

**Table 1:** Study inclusion and exclusion criteria

Study description	Inclusion criteria	Exclusion criteria
Design	Qualitative or mixed methods studies	Wrong Study Design: Mixed method studies with predominantly quantitative data
Aim/focus	Exploration of the psychological experiences of women with PCOS (including mental, emotional and/or psychosocial aspects)	Not Applicable: Studies focusing on the physiological aspects of PCOS without exploring psychological impact
Article type	Peer-review journal articles and conference papers with complete information	Not Applicable: Systematic reviews or narrative reviews, to prevent duplication of data.*
Participants	Women diagnosed with PCOS based on an established criteria such as the Rotterdam	Wrong Population: Women without a confirmed diagnosis of PCOS, or where the diagnosis was uncertain
Language	English	Foreign Language: Studies Published in languages other than English
Evidence	Subjective reports justified by participants excepts regarding their lived psychological experiences	Qualitative Studies with No Participant Excerpts: Accounts derived from recordings or observations of consultations by researchers, excluding direct quotations

Abbreviation: PCOS: polycystic ovary syndrome.

* While review papers were excluded to prevent duplication of data, papers were screened to identify any key studies that may have been overlooked. This approach helps to provide broad context to the existing literature and may highlight gaps in the current research.

### Critical appraisal

The primary researcher (FK) assessed the methodological quality of all included studies using the JBI Qualitative Assessment and Review Instrument (JBI-QARI) Critical Appraisal Checklist (Lockwood et al. 2015), which includes 10 "yes," "no," or "uncertain" questions. Studies with more than five "no" or "uncertain" responses were excluded. Those answering "yes" to all questions were graded "A," while those that did not were labelled "B." Table 2 presents the full assessment results.

**Table 2:** Critical appraisal of selected studies

Citation	Q1	Q2	Q3	Q4	Q5	Q6	Q7	Q8	Q9	Q10	Grade
1. Alsumri et al. (2023)	Y	Y	Y	Y	Y	Y	N	Y	Y	Y	**B**
2. *Amiri *et al*. (2014)*	Y	Y	Y	Y	Y	Y	N	Y	Y	Y	**B**
3. Atkinson et al. (2021)	Y	Y	Y	Y	Y	Y	N	Y	Y	Y	**B**
4. Authier et al. (2020)	Y	Y	Y	Y	Y	Y	N	Y	Y	Y	**B**
5. Copp et al. (2019)	Y	Y	Y	Y	Y	N	N	Y	Y	Y	**B**
6. Copp et al. (2019)	Y	Y	Y	Y	Y	N	N	Y	Y	Y	**B**
7. Ee et al. (2021)	Y	Y	Y	Y	Y	N	U	Y	Y	Y	**B**
8. Ekramzadeh et al. (2020)	Y	Y	Y	Y	Y	N	N	Y	Y	Y	**B**
9. Elghobashy et al. (2023)	Y	Y	Y	Y	Y	N	N	Y	Y	Y	**B**
10. Farajzadegan et al. (2023)	Y	Y	Y	Y	Y	N	N	Y	Y	Y	**B**
11. Hadjiconstantinou et al. (2017)	Y	Y	Y	Y	Y	N	N	Y	Y	Y	**B**
12. Hajivandi et al. (2022)	Y	Y	Y	Y	Y	Y	Y	Y	Y	Y	**A**
13. Holbrey and Coulson (2013)	Y	Y	Y	Y	Y	N	N	Y	Y	Y	**B**
14. Hopkins et al. (2019)	Y	Y	Y	Y	Y	N	N	Y	Y	Y	**B**
15. Hopkins et al. (2024)	Y	Y	Y	Y	Y	N	N	Y	Y	Y	**B**
16. Ismayilova and Yaya (2022)	Y	Y	Y	Y	Y	Y	N	Y	Y	Y	**B**
17. Ismayilova and Yaya (2022)	Y	Y	Y	Y	Y	N	N	Y	Y	Y	**B**
18. Ismayilova and Yaya (2023)	Y	Y	Y	Y	Y	Y	Y	Y	Y	Y	**A**
19. Jones et al. (2011)	Y	Y	Y	Y	Y	N	N	Y	Y	Y	**B**
20. Keegan et al. (2003)	Y	Y	Y	Y	Y	N	N	Y	U	Y	**B**
21. Lim et al. (2019)	Y	Y	Y	Y	Y	Y	N	Y	Y	Y	**B**
22. Saei Ghare Naz et al. (2019a)	Y	Y	Y	Y	Y	N	N	Y	Y	Y	**B**
23. Saei Ghare Naz et al. (2019b)	Y	Y	Y	Y	Y	N	N	Y	Y	Y	**B**
24. Percy et al. (2009)	Y	Y	Y	Y	Y	Y	N	Y	Y	Y	**B**
25. Pirotta et al. (2021)	Y	Y	Y	Y	Y	N	N	Y	Y	Y	**B**
26. Samardzic et al. (2021)	Y	Y	Y	Y	Y	Y	Y	Y	Y	Y	**A**
27. Taghavi et al. (2015)	Y	Y	Y	Y	Y	N	N	Y	Y	Y	**B**
28. Tay et al. (2021)	Y	Y	Y	Y	Y	Y	N	Y	Y	Y	**B**
29. Thorpe et al. (2019)	Y	Y	Y	Y	Y	Y	N	Y	Y	Y	**B**
30. Tomlinson et al. (2017)	Y	Y	Y	Y	Y	Y	N	Y	Y	Y	**B**
31. Wang et al. (2023)	Y	Y	Y	Y	Y	Y	N	Y	Y	Y	**B**
32. Weiss and Bulmer (2011)	Y	Y	Y	Y	Y	Y	N	Y	Y	Y	**B**
33. Williams et al. (2015)	Y	Y	Y	Y	Y	Y	N	Y	Y	Y	**B**
34. Wright et al. (2020)	Y	Y	Y	Y	Y	Y	Y	Y	Y	Y	**A**
35. Yin et al. (2022)	Y	Y	Y	Y	Y	Y	U	Y	Y	Y	**B**
36. Young et al. (2020)	Y	Y	Y	Y	Y	Y	N	Y	Y	Y	**B**
37. Nasiri Amiri et al. (2014)	Y	Y	Y	Y	Y	N	N	Y	Y	Y	**B**
38. Crete and Adamshick (2011)	Y	Y	Y	Y	Y	Y	N	Y	Y	Y	**B**
39. Kitzinger and Willmott (2002)	Y	Y	Y	Y	Y	Y	Y	Y	Y	Y	**A**
40. Pfister and Rømer (2017)	Y	Y	Y	Y	Y	Y	Y	Y	Y	Y	**A**
41. Snyder (2006)	Y	Y	Y	Y	Y	Y	N	Y	Y	Y	**B**
42. Williams et al. (2015)	Y	Y	Y	Y	Y	Y	Y	Y	Y	Y	**A**
43. Pathak (2021)	Y	Y	Y	Y	Y	Y	Y	Y	Y	Y	**A**

*Note*: Q1. Is there congruity between the stated philosophical perspective and the research methodology?

Q2. Is there congruity between the research methodology and the research question or objectives?

Q3. Is there congruity between the research methodology and the methods used to collect data?

Q4. Is there congruity between the research methodology and the representation and analysis of data?

Q5. Is there congruity between the research methodology and the interpretation of results?

Q6. Is there a statement locating the researcher culturally or theoretically?

Q7. Is the influence of the researcher on the research, and vice−versa, addressed?

Q8. Are participants, and their voices, adequately represented?

Q9. Is the research ethical according to current criteria or, for recent studies, and is there evidence of ethical approval by an appropriate body?

Q10. Do the conclusions drawn in the research report flow from the analysis, or interpretation, of the data?

Abbreviations: N, no; U, unclear; Y, yes

Of 43 studies appraised, eight (Grade A) met all criteria (Q1–Q10), ensuring rigorous methodology, ethical compliance, and robust data representation. The remaining 35 studies (Grade B) met most core criteria (e.g., congruity between methodology and research question, clear representation of participants, and ethical approval) but frequently lacked explicit cultural or theoretical positioning (Q6). These limitations are notable, as the absence of reflexivity reduces transparency regarding how the researchers’ perspectives may have affected data collection and/or interpretation. Whilst the data from Grade B studies are still considered relevant and credible, these quality gaps were considered when discussing the *Strengths and Limitations* section later on.

### Data extraction and synthesis

After database searches (see Supplementary File S2 for full search strategies), records were imported into EndNote 21, where initial duplicates were removed using the automated ‘Find Duplicates’ function, followed by manual verification.. The deduplicated library was uploaded to Rayyan®, where additional duplicates were flagged and reviewed. Rayyan ® was used for blinded title/abstract screening by three reviewers (FK, YT, NG), where each article was reviewed and coded as ‘include’, ‘exclude’, or ‘maybe’ with reasons provided. Conflicts and disagreements were resolved through discussions until consensus was achieved. Full-text screening of potentially eligible studies was also conducted in Rayyan® by the same three reviewers, following the same consensus process.

Data extraction was carried out using the JBI-QARI Standardized Data Extraction Tool Aromataris et al. 2024) to select data aligned with the research question. The primary researcher (FK) performed initial extraction and classified findings as unequivocal, credible, or unsupported. Two independent reviewers cross-checked data and quotations. Categories, subthemes, and themes were aggregated in Excel. The Biopsychosocial Model guided synthesis, framing interactions between biological symptoms, psychological responses, and sociocultural contexts. Thematic synthesis identified patterns across studies. Confidence in findings was assessed with the ConQual tool (Munn et al. 2014). All synthesis files, including extraction tables, are available on OSF (10.17605/OSF.IO/S6Y7N).

## Findings

### Study characteristics

Of 43 studies included, 38 were pure qualitative and five were mixed-method studies with detailed qualitative findings, collectively involving 1985 participants aged 13 to 63 years. All studies employed semi-structured interviews and focus group discussions, conducted in-person or telephonically. The studies spanned 10 countries, with the highest representation from the UK (10), followed by the US (7), and Australia (7), 5 Canada (5), Iran (7), China (2), and one each from Oman, France, Denmark, and India.

A total of 286 findings were extracted, each supported by participant quotes: 240 unequivocal and 46 credible. Findings were grouped into 11 categories and meta-aggregated into five themes (Table 3). This review discusses two themes in detail, with Table 3 showing how the 43 studies support each category and integrated themes. Each theme and subtheme is summarized with a brief synthesis of findings, accompanied by a participant excerpt, the study location and corresponding page number. Additional illustrative quotes can be found in the Supplementary Information (SS3).

**Table 3:** Major themes, subthemes, and subcategories

Major Themes	Subthemes	Subcategories
Theme One: Mental Health Challenges of PCOS	*Depression* *Anxiety* *Body Image Dissatisfaction*	Hopelessness; Perceived Loss of Control Fear of Infertility; Uncertainty & Long-Term Health Anxiety Unattractiveness & Low Self-Worth; Feeling “Less Feminine”
Theme Two: Psychosocial Challenges of PCOS	*Social Impacts* *Intimacy & Relationship Strain* *Sociocultural Variations*	Social Comparison; Societal Judgement & Stigma; Isolation
Theme Three: Psychological Impact of PCOS related to Diagnosis	*Diagnostic Challenges*	Diagnostic Delays; Medical Dismissal
Theme Four: Psychological Impact of PCOS related to Management	*Healthcare Barriers* *Lack of Information & Awareness*	Limited Access to Care; Limited Treatment Options; Lack of Support Misinformation & Lack of Knowledge
Theme Five: Coping with PCOS	*Active Coping Strategies* *Avoidant Coping Strategies*	Support networks; Self-education & Self-Advocacy; Acceptance & Resilience-Building Withdrawal & Avoidance; Humor as a Coping Mechanism

### Meta-synthesis of qualitative data

#### Synthesized finding 1: Mental health challenges

These synthesized findings demonstrate that the psychological impact of PCOS cannot be understood in isolation from its biological and sociocultural context. Women with PCOS frequently experienced depression, anxiety, and body image dissatisfaction, with some reporting them as isolated conditions, while others described them as co-occurring mental health challenges that impacted daily functioning (Hopkins et al. 2024).

*“I think the depression and anxiety have been the biggest issue I’ve had because it’s a showstopper. I couldn’t function for a while because of it.” (USA) (*Hopkins et al. 2024*) (p.191)*

Additional illustrative quotes supporting this theme and each corresponding subtheme are provided in Supplementary File S3, Theme 1: Mental Health Challenges (page 7).

#### Depression

Depression emerged as a prominent theme among women with PCOS (Alsumri et al. 2023; Ekramzadeh et al. 2020; Saei Ghare Naz et al. 2019a; Taghavi et al. 2015; Wright et al. 2020), with some stating that depression consumed their lives (Saei Ghare Naz et al. 2019a). PCOS-related depression is often linked to burdensome PCOS symptoms (Atkinson et al. 2021; Hopkins et al. 2024; Williams et al. 2015). An increase in body weight was repeatedly linked to embarrassment and depressive symptoms, fostering feelings of low self-worth (Lim et al. 2019). Similarly, hirsutism was reported as a major contributor to depression, as women struggled with its perceived impact on their self-image (Nasiri Amiri et al. 2014).

*“Having the PCOS adds to the depression because of all the things you hate about yourself.” (UK)(*Williams et al. 2015*)(p.05)*

*“”Invisible on the outside yet still visible in the eyes at times. Mental pain. Physical pain. Existential pain.” (Canada)(*Thorpe et al. 2019*)(p.03)*

Depression represented a disruption of a women’s sense of agency, identity, and everyday functioning. It emerged as strong mediating factor that undermines women’s motivation and capacity to engage in long-term management strategies, as well as their ability to sustain relationships, and maintain a positive self-perception.

#### Hopelessness

Hopelessness emerged as a prominent theme among women with PCOS, particularly in relation to its bothersome symptoms, infertility, and long-term health concerns (Alsumri et al. 2023; Farajzadegan et al. 2023; Saei Ghare Naz et al. 2019a; Taghavi et al. 2015). Infertility-related distress was especially linked to feelings of hopelessness, reinforcing the emotional burden associated with PCOS (Alsumri et al. 2023). Furthermore, many women experienced a deep sense of despair and an unfulfilled maternal identity, which in turn contributed to depression (Alsumri et al. 2023).

*“I felt like sitting somewhere alone and crying. Many women get pregnant; I think that I can’t get pregnant for the problem I have. I was very depressed because of that.” (Iran) (*Farajzadegan et al. 2023*)(p.394)*

Women described feeling consumed by their condition, with chronic symptoms and limited treatment options intensifying feelings of hopelessness (Ekramzadeh et al. 2020). This in turn led some women to resort to pharmaceutical interventions like antidepressants (Wright et al. 2020).

For some women, this emotional burden manifested in functional impairment and loss of interest in daily activities (Hopkins et al. 2024; Taghavi et al. 2015).

“*I am not interested in daily tasks*.” *(Iran)(*Pirotta et al. 2021*)(p.04)*

These narratives suggest that hopefully indicate that a perceived collapse of life possibilities, where normalcy and motherhood feel unattainable.

#### Perceived loss of control

The psychological burden of PCOS for some women outweighed its physical symptoms (Farajzadegan et al. 2023), with loss of control emerging as a key predictor of depression among participants with PCOS (Hopkins et al. 2024). Many women felt powerless over their bodies and emotions due to the unpredictability of PCOS symptoms (Farajzadegan et al. 2023; Hopkins et al. 2024; Wang et al. 2023; Williams et al. 2015). This intensified emotional distress, particularly when adequate medical guidance and support were lacking (Ekramzadeh et al. 2020). Furthermore, emotional instability in some severe depressive cases lead to suicidal ideation (Wang et al. 2023).

*“This disease resembles a brakeless car that makes you collapse either with diabetes or depression.” (Iran) (*Farajzadegan et al. 2023*)(p.395)*

These accounts suggest that when women with PCOS lose a sense of agency, their identity becomes destabilized and vulnerable, leaving them in a state of despair, and intensifying feelings of hopelessness and depression.

### Anxiety

Anxiety emerged as a prominent mental health challenge in women with PCOS, stemming from fear of infertility, uncertainty about PCOS and its long-term health implications (Alsumri et al. 2023; Copp et al. 2019; Weiss and Bulmer 2011). The unpredictability of symptoms, lack of control over fertility, and fear of chronic conditions contributed to persistent distress.

#### Fear of infertility and childlessness

Infertility-related anxiety emerged as a key contributor to anxiety in women with PCOS, particularly for those struggling to conceive, as many viewed motherhood as central to their identity (Alsumri et al. 2023; Copp et al. 2019; Hadjiconstantinou et al. 2017; Wang et al. 2023).

*“… I wish I can have a child and I hear someone calling me ‘Mama’...I’m afraid I’ll die before I get to experience this” (Oman) (*Alsumri et al. 2023*)(p.111)*

For adolescents with PCOS, this anxiety began early, as they feared that infertility could limit their chances of future relationships (Jones et al. 2011; Saei Ghare Naz et al. 2019a). Notably, some women considered adoption to avoid the distress of failed pregnancies (Weiss and Bulmer 2011).

*“When it’s time, I’m a little scared my dream is to adopt I just don’t think it’s necessary to go through all the heartache of trying to have babies and not be successful.”* (USA) (Weiss and Bulmer 2011) (p.712)

Desperation over fertility treatments often lead to short-term solutions over sustainable approaches (Pirotta et al. 2021), with anxiety worsening as older women face more invasive treatments (Taghavi et al. 2015). Infertility also contributed to unhealthy coping mechanisms to provide emotional relief, such as eating disorders (Nasiri Amiri et al. 2014).

*“…I'm always stressed. In terms of how I manage it… I tend to resort to quick fixes.” (Australia*^*57*^*) (p.2048)*

This demonstrates how PCOS manifestations often content with sociocultural ideals of womanhood, where reproductive ability is equated with value and identity.

#### Uncertainty and long-term health anxiety

Uncertainty about symptoms, fertility, treatment efficacy, and long-term health emerged as a major source of anxiety for women with PCOS (Authier et al. 2020; Copp et al. 2019; Farajzadegan et al. 2023; Nasiri Amiri et al. 2014; Saei Ghare Naz et al. 2019b; Snyder 2006; Yin et al. 2022). This distress was particularly pronounced in adolescents, who struggled with the lifelong nature of PCOS and the need for continuous management (Saei Ghare Naz et al. 2019b).

*“… What will happen? When there is no treatment, I will be sick until the end of my life. What should I do?” (Iran) (*Farajzadegan et al. 2023*)(p.394)*

Furthermore*,* uncertainty surrounding PCOS symptoms such as weight fluctuations, unpredictable menstrual irregularities, and reproductive challenges further exacerbated feelings of helplessness and anxiety (Snyder 2006; Yin et al. 2022).

*"I just don’t know when I will have menstruation or when I will be pregnant. This is the uncertainty" (China) (*Yin et al. 2022*) (p.300)*

Women with PCOS experienced long-term health anxiety, fearing disease progression and its broader health implications (Copp et al. 2019; Weiss and Bulmer 2011). Noteworthy, adolescents frequently expressed distress over the probable worsening of ovarian cysts (Ekramzadeh et al. 2020) and the increased risk of developing ovarian cancer (Young et al. 2020). Fears of developing chronic conditions such as diabetes, cardiovascular disease and hypertension further contributed to anxiety and distress (Crete and Adamshick 2011; Farajzadegan et al. 2023; Thorpe et al. 2019).

*“I cried because of the difficulties of the condition and eventually what could happen with my developing diabetes and heart problems.” (USA) (*Crete and Adamshick 2011*)(p.262)*

These accounts reveal that anxiety in this context, represents a hyper-vigilant and anticipatory state focused on health outcomes, personal and social expectations, and symptom progression. This obsessive preoccupation with future uncertainties undermines women’s ability to take control and make better choices to manage their condition, ultimately exacerbating the psychological burden of the disease.

### Body image dissatisfaction

#### Unattractiveness & low self-worth

Women with PCOS commonly reported feeling unattractive and physically inferior due to their symptoms, leading to negative self-perception and low self-worth (Weiss and Bulmer 2011). Women struggled with body dysmorphia and the fear of being perceived as abnormal or a "freak" (Kitzinger and Willmott 2002; Wright et al. 2020). A strong preoccupation with symptoms like acne, oily skin, hair thinning, and weight gain reinforced feelings of unattractiveness, embarrassment, and low self-esteem, often leading to social withdrawal (Ekramzadeh et al. 2020; Elghobashy et al. 2023; Hopkins et al. 2019; Nasiri Amiri et al. 2014; Saei Ghare Naz et al. 2019a; Yin et al. 2022).

*“I have horrible self-esteem because of the way my body is. I am very ashamed of the weight and acne and thinning hair.” (USA)(*Wright et al. 2020*)(p.1731)*

Many overweight women opted for concealing clothing to minimize feelings of inferiority while rapid weight fluctuations and acne heightened self-consciousness and distress (Hadjiconstantinou et al. 2017; Hopkins et al. 2019; Nasiri Amiri et al. 2014; Saei Ghare Naz et al. 2019b; Thorpe et al. 2019; Wang et al. 2023; Yin et al. 2022; Young et al. 2020). Despite significant efforts, many struggled with weight loss, fostering self-doubt and inadequacy (Lim et al. 2019; Pirotta et al. 2021).

*"I am anxious about my body image, as my dress style is now limited. I no longer wear skirts or shorts, instead opting for loose pants or long skirts. This makes me feel inferior to others." (China) (*Wang et al. 2023*) (p.04)*

Hirsutism further intensified embarrassment and unfavourable self-comparisons, leading to social withdrawal and persistent shame (Crete and Adamshick 2011; Hadjiconstantinou et al. 2017; Jones et al. 2011; Keegan et al. 2003; Kitzinger and Willmott 2002; Wright et al. 2020).

*“The hairy face is unusual and hence ‘‘unnatural’’ for a woman. And ugly.” (UK) (*Kitzinger and Willmott 2002*) (p.354)*

#### Feeling “Less Feminine”

Women felt less feminine due to the physical and reproductive symptoms of PCOS, with hirsutism being the most distressing. Many described it as "unnatural," and “freakish,” intensifying self-consciousness and identity loss (Keegan et al. 2003; Snyder 2006; Williams et al. 2016). Women attempted to conceal facial hair with scarves or hair removal treatments, but frustration grew when these efforts proved unsustainable (Taghavi et al. 2015; Williams et al. 2016). Feelings of masculinity was often reinforced by intimate partners through comments about their appearance or behaviour (Amiri et al. 2014b), as well as the use of masculine descriptors such as “stache and goatee” (Williams et al. 2016). Women in relationships felt pressured to conform to conventional femininity, yet unpredictable PCOS symptoms made this challenging (Taghavi et al. 2015).

*“I feel more masculine... For example, he (my husband) always told me that ‘you mainly behave manly, it seems like you are not a woman and your masculine tempers are more." (Iran)(*Amiri et al. 2014b*)(p.05)*

Acne, increased body mass, and alopecia also reinforced feelings of lost femininity, with some women believing PCOS had "ruined" their bodies (Thorpe et al. 2019; Williams et al. 2016). Menstrual irregularities, particularly the absence of menstruation, contributed to feelings of abnormality (Kitzinger and Willmott 2002; Williams et al. 2016). Additionally, some women equated infertility with a loss of womanhood, finding it even more distressing than the physical symptoms of PCOS (Amiri et al. 2014b; Kitzinger and Willmott 2002).

*" I feel that if I can't bear a child; I will lose all sense of being a woman.” (Iran) (*Amiri et al. 2014b*)(p.05)*

These findings highlight that depression, anxiety, and body image dissatisfaction were not isolated mental health challenges linked solely to specific PCOS symptoms, but interdependent factors shaped by broader psychosocial dynamics. Social comparison, stigma, cultural and societal expectations often mediated the severity and expression of these mental health challenges. Furthermore, the unpredictable and chronic nature of PCOS impaired women’s sense of agency and self-worth, and in turn disrupted their anticipated lifepaths such as motherhood, marriage, and stable health. This dissonance between the lived reality of women with PCOS and their anticipated lifepath suggests that PCOS is experienced not only as a physiological condition, but also a psychosocial and existential burden.

### Synthesized finding 2: Psychosocial challenges

Additional illustrative quotes supporting this theme and each corresponding subtheme are provided in Supplementary File S3, Theme 2: Psychosocial Challenges (page 7–8).

#### Social comparison

Social comparison emerged as a mechanism through which women internalized sociocultural ideals of womanhood, leading to pressure to conform to these ideals. Women with PCOS frequently compared themselves to others which often led to depression, self-blame, and social withdrawal (Amiri et al. 2014b). Infertility emerged as a major factor that led women to compare themselves to those who conceived easily, evoking feelings of inadequacy, envy, and resentment (Abdolvahab Taghavi et al. 2015; Alsumri et al. 2023; Taghavi et al. 2015; Thorpe et al. 2019; Wright et al. 2020). Some women internalized blame for their infertility, particularly when comparing themselves to family members who conceived easily (Amiri et al. 2014b; Crete and Adamshick 2011).

*“Every single wife that is married and their husband are in the same unit are all having babies.” (USA) (*Wright et al. 2020*)(p.1731)*

*“When I see a pregnant woman or those with kids, I envy so much that I want to kill them.” (Iran) (*Taghavi et al. 2015*) (p.05)*

These accounts suggest that social comparison was often shaped by women’s immediate social environments, including neighbours, family members, and peers. In such sociocultural contexts where childbearing was expected of young married couples, such comparisons intensified social pressure and deepened women’s internalised feelings of failure and low self-worth.

#### Societal judgement and stigma

Women encountered societal judgment and stigma due to their symptoms, which conflicted with dominant beauty standards and cultural expectations (Elghobashy et al. 2023; Keegan et al. 2003; Thorpe et al. 2019). The pressure to conform to idealized norms such as a slim figure, and minimal body hair, led to distress, self-consciousness, and isolation (Elghobashy et al. 2023; Hadjiconstantinou et al. 2017; Wright et al. 2020).

*“I find that the pressures imposed by society on women to be thin and have beautiful hair are in direct opposition with the symptoms I have experienced as a result of PCOS.” (UK)(* Elghobashy et al. 2023)*(p.05)*

Women endured intrusive and persistent questions about their fertility (Pathak 2021; Taghavi et al. 2015; Yin et al. 2022), causing some to conceal their concerns to avoid stigma (Kitzinger and Willmott 2002). Hirsutism was another major source of public humiliation, with many women experiencing bullying, embarrassment, and isolation (Pfister and Rømer 2017; Samardzic et al. 2021; Tay et al. 2021; Wright et al. 2020; Yin et al. 2022).

*“They are always asking me why I did not plan to get pregnant? What seems to be the problem?” (Iran)(*Taghavi et al. 2015*)(p.05)*

Acne heightened self-consciousness, prompting some women to conceal their skin or avoid eye contact to minimize unwanted attention (Hadjiconstantinou et al. 2017). Weight stigma and criticism were also prevalent (Nasiri Amiri et al. 2014; Weiss and Bulmer 2011). leading some women to avoid activities like outdoor exercise due to fear of judgment, further impacting symptom management (Lim et al. 2019).

*"You know you’re overweight, you have hair growing where you don’t normally, and you get bullied and picked on… I missed out on a lot of opportunities that other people may have been able to easily obtain, like relationships and friendships." (Australia)(*Tay et al. 2021*)(p.04)*

A lack of awareness further fuelled PCOS-related stigma, with some women frustrated by its dismissal as an excuse for weight gain rather than a legitimate condition (Thorpe et al. 2019), which worsened isolation and made it difficult for women to self-advocate (Hadjiconstantinou et al. 2017; Hopkins et al. 2019; Keegan et al. 2003; Nasiri Amiri et al. 2014; Saei Ghare Naz et al. 2019a). Many women avoided discussions about PCOS to prevent burdening others and thus internalized distress (Snyder 2006; Young et al. 2020).

“Yeah, you don’t want to bum everyone out.” (USA)(Young et al. 2020) (p.07)

In some cases, women experienced discrimination and public humiliation, particularly in occupations with strict appearance standards, such as the military (Hopkins et al. 2024).

*“I had a platoon sergeant walk up to me, he asked me if I had a shaving profile, I may need one or want to look into it. And this was called out in front of a group of at least 50 people.” (USA)(*Hopkins et al. 2024*)(194)*

These narratives indicate that stigma exacerbated psychological distress by positioning PCOS symptoms in direct contrast to cultural ideals of beauty, particularly Western beauty standards, and womanhood.

#### Social Isolation

Women frequently reported experiencing isolation due to the stigma, judgment, and emotional burden associated with the condition (Authier et al. 2020; Snyder 2006).

#### *"I feel so isolated, like I’m the only one that has this." (USA)(*Snyder 2006*) (p.388)*

Women expressed feeling different from their peers, leading to a sense of exclusion and abnormality (Hopkins et al. 2019). The emotional toll of navigating PCOS often led to withdrawal from meaningful social interactions, with some women selectively confiding to avoid judgement (Hadjiconstantinou et al. 2017; Keegan et al. 2003; Nasiri Amiri et al. 2014; Saei Ghare Naz et al. 2019b; Snyder 2006; Young et al. 2020). Isolation was evident in women who struggled with body image concerns, as embarrassment over physical appearance led to avoidance of activities, further hindering symptom management (Lim et al. 2019).

*“Often exercise I don't want to do it outside of the house at all. I don't want people to see me even walking down the street.” (Australia)(*Lim et al. 2019*)(p.05)*

Isolation thus emerges as a compounding factor that worsens the cycle of mental health distress by cutting women off from support systems that could buffer psychological strain. By retreating, women attempt to shield themselves from judgement in an attempt to preserve their mental health, but this also isolates them from potential sources of support, amplifying depression and anxiety.

#### Intimacy and Relationship Strain

PCOS significantly impacted intimate relationships, with infertility and body image concerns leading to emotional disconnection between partners (Alsumri et al. 2023). Infertility caused marital tension, frustration, and resentment, deepening women's sense of isolation (Nasiri Amiri et al. 2014). Hirsutism and weight gain further contributed to intimacy challenges, as many women felt unattractive and undeserving of love (Pfister and Rømer 2017; Thorpe et al. 2019).

*“Yes, it affected us so much. I can feel that we became less connected with each other”. (Oman) (*Alsumri et al. 2023*)(p.111)*

*“I have always felt that I should be grateful that any man wants to be with me… with my spots and hairs and obesity.” (Canada)(*Wang et al. 2023*) (p.04)*

Sexual dissatisfaction and loss of desire were common, with some women avoiding intimacy due to self-consciousness and embarrassment over their symptoms (Abdolvahab Taghavi et al. 2015; Jones et al. 2011; Taghavi et al. 2015). Fertility struggles also diminished sexual interest, as some viewed intercourse as futile (Taghavi et al. 2015). In severe cases, marital conflicts escalated, with women facing accusations of infertility from their spouses (Nasiri Amiri et al. 2014).

*“He said, ‘you are infertile’, and therefore, he wants a separation.” (Iran) (*Amiri et al. 2014a*) (p.05)*

These accounts highlight how PCOS destabilizes relational bonds by undermining both partners’ sense of intimacy, reinforcing women’s perception of themselves as “unworthy” or “unlovable” partners.

### Sociocultural variation in psychosocial challenges

Cultural factors played a significant role in shaping the psychosocial challenges faced by women with PCOS. Women from countries such as the UK, USA, Canada, and Australia, reported distress related to Western beauty standards, which emphasized being thin and hairless. These cultural ideals magnify psychological symptoms such as body dissatisfaction, anxiety, and low self-worth, showing how distress over acne, hirsutism, or weight gain is not only physical but socially mediated (Elghobashy et al. 2023; Hadjiconstantinou et al. 2017; Keegan et al. 2003; Thorpe et al. 2019; Wright et al. 2020).

*"I mean I come from (place name in the USA) where it’s absolutely taboo to walk around with leg hair, or underarm hair" (UK) (*Keegan et al. 2003*)(p.337)*

Conversely, in Asian, Middle Eastern, and Islamic cultural contexts such as in Iran, Oman, and India, infertility and marital conflict emerged as dominant concerns due to the centrality of marriage and childbearing (Abdolvahab Taghavi et al. 2015; Alsumri et al. 2023; Nasiri Amiri et al. 2014; Taghavi et al. 2015). Women faced intense familial and societal pressure, intrusive questioning, and blame over infertility, often leading to familial conflict and marital strain (Elghobashy et al. 2023; Hadjiconstantinou et al. 2017; Keegan et al. 2003; Saei Ghare Naz et al. 2019a; Thorpe et al. 2019; Wright et al. 2020). Further sociocultural distinctions were observed in India, where traditional gender roles and familial expectations around food hindered PCOS management. Further sociocultural distinctions emerged in Indian contexts, where familial and societal expectations around food and traditional gender roles hindered women’s ability to manage their PCOS. Additionally, prolonged festivities posed difficulties in maintaining dietary discipline, with social events creating pressure to indulge in traditional foods (Pathak 2021).

*“She can’t cook a healthy meal. If she cooks a salad, the mother-in-law says ‘You are starving my son!” (India) (*Pathak 2021*) (p330)*

These cross-cultural distinctions demonstrate how psychological symptoms and sociocultural expectations interrelate, depending on which sociocultural expectation is dominant. In Western contexts, body image distress aligns with beauty ideals; in Middle Eastern and South Asian contexts, infertility and marital strain dominate. This demonstrates that psychological distress is not experienced in isolation but is mediated by the cultural lens through which womanhood is defined.

**Fig. 2:**
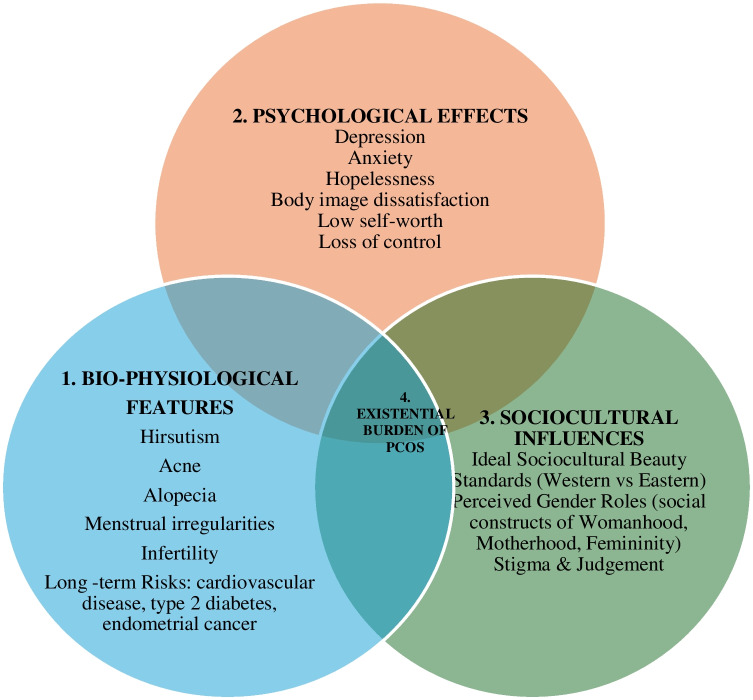
Biopsychosocial Model of PCOS. 1.Bio-Physiological Features: Clinical manifestations of PCOS act as primary stressors that trigger psychological and social challenges. 2.Psychological Effects: These symptoms frequently lead to depression, anxiety, body image dissatisfaction, hopelessness, and reduced self-worth, which undermine women’s sense of agency and everyday functioning. 3.Sociocultural Influences: Cultural norms and societal expectations, particularly those linking femininity to appearance and motherhood, intensify distress through stigma, judgment, and relationship strain. 4. Intersection: The interplay between these domains illustrates how biological symptoms of PCOS acquire meaning through cultural lenses, shaping psychological responses in ways that vary across age, life stage, and sociocultural context. This framework guided synthesis by integrating findings beyond symptom descriptions, highlighting how PCOS functions as not only a medical condition but also a psychosocial and existential burden.

Collectively, these findings illustrate that the psychological burden of PCOS emerges at the intersection its bio-physiological symptoms, mental health challenges, and sociocultural norms and expectations. Figure 2 illustrates these interconnections with a biopsychosocial framework.

While the primary analysis focused on mental health and psychosocial challenges, supplementary findings also highlight diagnostic delays, management dissatisfaction, and the importance of coping strategies (see Supplementary Information SS4). The ConQual method (Munn et al. 2014) was used to assess the dependability and credibility of these synthesized findings. The ConQual summary of findings is shown in Table 4 below.

**Table 4:** ConQual summary of findings

**Synthesised findings**	**Type of research**	**Dependability**^1^	**Credibility**^2^	**ConQual**^**3**^**score**	**Comments**
**Mental Health Challenges of PCOS** include depression, anxiety, and body image dissatisfaction. The unpredictability of symptoms contributes to a perceived loss of control, while fear of infertility and long-term health uncertainty exacerbate distress. Body image concerns, particularly unattractiveness and feeling "less feminine," negatively impact self-worth and emotional well-being	Qualitative	Downgrade 1 level*	High (remains unchanged)	Moderate	Multiple participant quotes across studies present similar findings Out of 98 findings extracted from 44 studies, the majority of findings (95) were unequivocal and 3 were credible Although Jones et al. (2024) provided credible findings, supporting quotes were fragmented, referencing specific words/phrases from multiple participants rather than complete, standalone statements
**Psychosocial Burden of PCOS** PCOS significantly affects social relationships, leading to social comparison, judgment, stigma, and isolation. Visible symptoms, such as weight gain, acne, and hirsutism, result in public scrutiny and emotional distress. Women face challenges in intimate relationships, with infertility and body image concerns contributing to emotional disconnection, reduced libido, and relationship strain	Qualitative	Downgrade 1 level*	Downgrade 3 levels	Moderate	Many quotes throughout studies support findings Out of 40 findings extracted from 18 studies, the majority of findings (37) were unequivocal, 3 findings were credible, 1 finding was unsupported Despite a few credible and one unsupported finding, most findings were unequivocal, warranting a moderate ConQual rating for this synthesized finding
**Sociocultural variation** shapes the psychosocial impact of PCOS, influencing beauty standards, infertility distress, and workplace stigma	Qualitative	Downgrade 1 level*	Downgrade 2 levels	Low	Limited quotes supported the findings Out of 7 findings extracted from 7 studies, 6 were unequivocal, 1 was credible

1Is there congruity between the research methodology and the research question or objectives?

Is there congruity between the research methodology and the methods used to collect data?

Is there congruity between the research methodology and the representation and analysis of data? Is there a statement locating the researcher culturally or theoretically?

Is the influence of the researcher on the research, and vice−versa, addressed?

2Unequivocal (findings accompanied by an illustration that is beyond reasonable doubt and; therefore not open to challenge, 0 or −1 level)

Credible (findings accompanied by an illustration lacking clear association with it and therefore open to challenge, −2 levels)

Unsupported (findings are not supported by the data, −3 levels)

3High, Moderate, Low, Very Low

*Downgraded one level due to prevalent dependability concerns across the included primary studies. While 25 studies provided a statement locating the researcher culturally or theoretically, 18 did not, and only 6 acknowledged the researcher's influence on the study

## Discussion

To strengthen integration of findings, the Biopsychosocial Model provided a useful interpretive lens, illustrating how biological manifestations of PCOS (e.g., hirsutism, weight gain, infertility) intersect with women’s psychological responses (e.g., depression, anxiety, body image dissatisfaction) and sociocultural expectations (e.g., pressure to conceive, stigma around femininity). Whilst the clinical features of PCOS act as significant psychological trigger of PCOS, the findings clearly show that the meaning and intensity of this distress were shaped by the sociocultural lens through which these symptoms were experienced.

Bio-physiological symptoms of PCOS perpetually undermined women’s sense of femininity and normalcy. Hirsutism, weight gain, acne, and alopecia were strongly associated with depression, body dissatisfaction, and diminished self-worth, while infertility disrupted life trajectories centred on marriage and motherhood (Atkinson et al. 2021; Hopkins et al. 2024; Williams et al. 2015). The unpredictability of symptoms often created a sense of loss of control, which intensified hopelessness and reduced women’s ability to plan for the future (Farajzadegan et al. 2023; Wang et al. 2023). These findings suggest that the clinical features of PCOS act as catalysts for broader psychosocial strain.

Depression, anxiety, and body image dissatisfaction emerged as prominent and interdependent mental health challenges, shaped largely by sociocultural expectations. Infertility-related anxiety in particular was intensified in settings where motherhood was attributed to womanhood and femininity. Moreover, visible PCOS symptoms such as facial hair, alopecia, and acne triggered shame and feelings of low self-worth in societies where these features were symbolized as being “masculine”, thereby destabilizing women’s gender identity (Keegan et al. 2003; Wright et al. 2020). Psychological distress varied across the life course. In adolescence, acne and facial hair heightened vulnerability during identity formation and peer comparison. In early adulthood, fertility struggles, and uncertainty overshadowed other concerns, while by midlife, fears of chronic conditions such as diabetes and cardiovascular disease became dominant. These shifts show that PCOS-related distress evolves with age, shaped by both biological changes and sociocultural expectations. Social comparison, stigma, and marital strain reinforced the mental health challenges faced by women with PCOS. Infertility, specifically, positioned women in constant comparison to fertile peers, triggering feelings of inadequacy, envy, and failure (Alsumri et al. 2023; Taghavi et al. 2015). Public stigma around weight, acne, and hirsutism further fuelled isolation, with some women concealing their condition or withdrawing from relationships to avoid judgment (Thorpe et al. 2019; Young et al. 2020). Whilst social withdrawal intended to shield women from judgement, it simultaneously deprived them of support systems that could help them cope with PCOS, thereby exacerbating mental health impacts. Furthermore, relationship strain was common, as partners sometimes reinforced negative self-perceptions by questioning femininity or attributing marital instability to infertility. These sociocultural dynamics magnified the psychological toll of PCOS, reinforcing that symptoms acquire meaning through social contexts rather than in isolation. This synthesis clearly highlights that the lived experiences of PCOS are inseparable from the sociocultural scripts of femininity, motherhood, and idealized beauty standards. Depression, anxiety, and body dissatisfaction cannot be addressed in isolation from the social comparisons, stigma, and marital expectations that amplify them. The findings of this review have important clinical implications that extendbeyond biomedical symptom management. The common PCOS manifestations must beconsidered as both physiological concerns and psychological stressors. This implies that routinemental health screening for depression, anxiety, and body image dissatisfaction should beintegrated into PCOS care, with particular attention to adolescence and fertility planning. Inaddition, culturally sensitive, non-stigmatizing mental health counselling that addresses fertility,femininity, and long-term health risks across all levels of care is essential to mitigatepsychological distress and prevent the worsening of both mental health and clinical outcomes.General practitioners and nurses are integral first points of healthcare contact in the earlyrecognition of PCOS-related symptoms. This will assist in initiating timely referrals for diagnosticinvestigations, including ultrasound assessment and specialist gynaecological care. Ongoingtraining in PCOS education across primary and specialist healthcare services are thereforeessential to establish clearly defined referral pathways, standardised screening practices, andeffective communication from first contact through to specialist care. This findings from thisreview further underscores the value of interdisciplinary management systems involvingprimary care providers, gynaecologists, endocrinologists, and mental health professionals.Collectively, these integrated approaches may improve both physical and mental healthoutcomes and promote more person-centred care for women living with PCOS.

### Strengths and limitations

This SR strengthens existing PCOS literature by providing a comprehensive analysis of its mental, emotional, and psychosocial challenges, rather than focusing on isolated aspects. By synthesizing diverse experiences, it offers a holistic understanding of the psychological burden of PCOS. Incorporating the most recent research, it captures contemporary perspectives while accounting for sociocultural variations. A rigorous methodological approach ensures alignment between research design, data collection, and analysis, enhancing the reliability of findings.

A key limitation, however, was that only 8 of the 43 included studies received an “A” grading on the JBI-QARI checklist, with the majority graded “B” due to insufficient reflexivity and theoretical positioning. These gaps limit insight into how researchers’ perspectives, cultural standpoints, or methodological assumptions may have affected data interpretation.. Whilst the findings still remain credible and relevant, their transferability to all cultural and clinical settings must be interpreted with caution. Furthermore, the lack of reflexivity may potentially reduce credibility, as it makes it more difficult to fully evaluate researcher bias.

Additional limitations should also be noted. First, only English-language studies were included, which may have excluded relevant research published in other languages and introduced language bias. Second, the exclusion of grey literature (e.g., theses, dissertations, and non-indexed reports) raises the possibility of publication bias, as studies with null or divergent findings may be underrepresented. Third, there is an underrepresentation of studies from low-income and non-Western contexts. This imbalance could not be avoided, as few studies from these settings met the inclusion criteria. The absence of such perspectives highlights the urgent need for more PCOS research in diverse sociocultural and economic contexts, which would provide a more globally representative understanding of women’s lived experiences.

Despite these constraints, the cross-study convergence of participant experiences strengthens dependability and supports the overall trustworthiness of the synthesis.

## Conclusion

This SR highlights the significant psychological burden experienced by women with PCOS, with depression, anxiety, body image dissatisfaction, and social stigma emerging as dominant challenges. Notably, hirsutism, menstrual irregularities, weight gain, acne, and infertility deeply affect female identity and well-being, while sociocultural factors, such as Western beauty ideals and cultural emphasis on childbearing in Middle Eastern and South Asian contexts, further intensify distress. Based on our findings, we recommend three priority actions for practice and policy:

1. **Routine mental health screening**: Incorporating brief, validated tools such as the PHQ-9 (for depression) and GAD-7 (for anxiety) into standard PCOS care to identify at-risk women early.
2. **Culturally and age-sensitive psychological interventions**: Prioritizing access to evidence-based therapies such as cognitive-behavioural therapy (CBT), adapted for cultural context, and supplemented by peer support groups.
3. **Integrated fertility education and counselling**: Providing clear, supportive fertility guidance early in the care pathway to reduce uncertainty, stigma, and anxiety.

At the policy level, these strategies should be embedded within PCOS management guidelines to ensure holistic care that addresses both physical and psychological needs. Future research should focus on evaluating the effectiveness of such culturally tailored interventions across diverse populations and life stages. By prioritizing screening, accessible interventions, and culturally sensitive care, clinicians and policymakers can reduce the psychological burden of PCOS and improve QoL for affected women.

## Supplementary Information

Below is the link to the electronic supplementary material.

Supplementary file1 (DOCX 351 KB)

## Data Availability

The JBI-QARI data extraction and analysis files supporting this review are available on OSF (templates available at: https://osf.io/s6y7n/files/osfstorage or via the 10.17605/OSF.IO/S6Y7N).
