# Supplementary material for: The lived experiences of women with polycystic ovary syndrome and its psychological challenges: A systematic review and meta-synthesis
Source: Arch Womens Ment Health. 2026 Feb 12;29(1):39. doi: 10.1007/s00737-025-01636-4 (PMC12894167; doi:10.1007/s00737-025-01636-4)
Supplement: Supplementary file 1 — Supplementary file1 (DOCX 351 KB) [file 737_2025_1636_MOESM1_ESM.docx]

**Supplementary Information for** **“*The Lived Experiences of Women with Polycystic Ovary Syndrome and its Psychological Challenges: A Systematic Review and Meta-Analysis*”**

Faathimah Khan^1^, Nalini Govender^2^ (PhD), Yasmeen Thandar^3^ (PhD), Sara Bibi Mitha^4^

^1,2,3^ Dept of Basic Medical Sciences, Durban University of Technology, Durban, South Africa

^4^ Alan Pittendrigh Library, Steve Bhiko Campus, Durban University of Technology, South Africa

Corresponding author email: [Faathimah.nkhan@gmail.com](mailto:Faathimah.nkhan@gmail.com)^1^

Journal Name: Archives of Women’s Mental Health

**Contents**

[Supplementary Section 1 (S1): Socio-demographic variation among PCOS patients 2](#_Toc209529463)

[Supplementary Section 2 (S2): Search Strategy Used in Systematic Review Methodology 3](#_Toc209529464)

[Supplementary Section 3 (S3): Illustrative Quotes 7](#_Toc209529465)

[Supplementary Section 4 (S4): Additional Synthesized Findings 9](#_Toc209529466)

[Supplementary Information Section 5 (S5): ConQual summary of findings 10](#_Toc209529467)

## Supplementary Section 1 (S1): Socio-demographic variation among PCOS patients

Alur-Gupta et al.^29^ found Black women with PCOS in the US reported lower Q scores than White women. Despite higher generalized anxiety disorder prevalence in White women, Black women reported lower anxiety, possibly due to resilience, social support, and substance abuse disparities. White women had higher body image concerns, while Black women were less likely to seek help for sexual health issues due to socio-economic and cultural influences. These disparities underscore the role of ethnicity in the psychosocial well-being of women with PCOS^34^. Other factors like age, relationship status, educational level and profession remain underexplored^31-33^, but warrant consideration ^35^. Larger, well-controlled studies are needed to explore these associations.

## Supplementary Section 2 (S2): Search Strategy Used in Systematic Review Methodology

Date: 1 July 2024

**Search terms. Search in Title/ keyword/ abstract**

| **Topic** | **Mesh Heading** |
| --- | --- |
| “Polycystic ovarian syndrome” OR “polycystic ovary syndrome” OR “stein-Leventhal syndrome” OR PCOS OR PCO | **Polycystic Ovary Syndrome**  ((polycystic ovarian syndrome[MeSH Terms]) OR (polycystic ovary syndrome[MeSH Terms])) OR (syndrome, polycystic ovary[MeSH Terms]) |
| **AND** |  |
| **Topic** | **Mesh Heading** |
| “Lived experience*” OR **"**individual experience*" OR "personal experience*" OR "first hand experience*" OR "first-hand account" OR "personal narrative" OR "firsthand knowledge" OR "personal perspective" OR "real-life experience" OR "direct experience" OR "experiential knowledge" OR "authentic experience" OR interview* OR opinion OR qualitative OR “mixed methods” OR “action research" OR "feminist research" OR "ground theory" OR phenomenology | Health services research OR personal narratives OR interviews, psychological |

**AND**

| **Topic** | **Mesh Heading** |
| --- | --- |
| Psychological OR “psychological health” OR “psychological well-being” OR “Mental health” OR “mental health disorder” OR “mental health challenges” OR “mental well-being” OR “mental illness” OR “psychiatric disorder” OR “mood disorder” OR “eating disorders” OR “sleep disorders” OR anxiety OR depression OR“ Emotional disorders” OR “emotional challenges” OR “emotional well-being” OR “emotional problems” OR “emotional distress” OR “Psychosocial disorders” OR “psychosocial challenges” OR “psychosocial well-being” OR “psychosexual difficulties” OR “social isolation” OR “social anxiety” OR “self-esteem” OR “self-image” OR “body image” OR “sexual functioning” OR “sexual disorder” | Stress, psychological OR anxiety disorder OR psychological well-being OR mental disorder OR self concept OR mood disorders OR dyssomnias  ((stress, psychological[MeSH Terms]) OR (anxiety disorder[MeSH Terms])) OR (anxiety disorders[MeSH Terms])) OR (psychological well-being[MeSH Terms])) OR (mental disorder[MeSH Terms])) OR (self concept[MeSH Terms])) OR (mood disorder[MeSH Terms])) OR (mood disorders[MeSH Terms])) OR (dyssomnia[MeSH Terms])) OR (dyssomnias[MeSH Terms]) |

**Web of Science (1994 to present)**

| **“Polycystic ovarian syndrome” OR “polycystic ovary syndrome” OR “stein-Leventhal syndrome” OR PCOS or PCO** (Topic) and **“Lived experience*” OR "individual experience*" OR "personal experience*" OR "first hand experience*" OR "first-hand account" OR "personal narrative" OR "firsthand knowledge" OR "personal perspective" OR "real-life experience" OR "direct experience" OR "experiential knowledge" OR "authentic experience" OR interview* OR opinion OR qualitative OR "mixed methods" OR “action research" OR "feminist research" OR "ground theory" OR phenomenology** (Topic) and **Psychological OR “psychological health” OR “psychological well-being” OR “Mental health” OR “mental health disorder” OR “mental health challenges” OR “mental well-being” OR “mental illness” OR “psychiatric disorder” OR “mood disorder” OR “eating disorders” OR “sleep disorders” OR anxiety OR depression OR“ Emotional disorders” OR “emotional challenges” OR “emotional well-being” OR “emotional problems” OR “emotional distress” OR “Psychosocial disorders” OR “psychosocial challenges” OR “psychosocial well-being” OR “psychosexual difficulties” OR “social isolation” OR “social anxiety” OR “self-esteem” OR “self-image” OR “body image” OR “sexual functioning” OR "sexual disorder"** (Topic) and **English** (Languages) and **Meeting Abstract** (Exclude – Document Types) |
| --- |

Results = 126

**SCOPUS (1996 to present)**

| ( TITLE-ABS-KEY ( "Polycystic ovarian syndrome" OR "polycystic ovary syndrome" OR "stein-Leventhal syndrome" OR pcos OR pco ) AND TITLE-ABS-KEY ( "Lived experience*" OR "individual experience*" OR "personal experience*" OR "first hand experience*" OR "first-hand account" OR "personal narrative" OR "firsthand knowledge" OR "personal perspective" OR "real-life experience" OR "direct experience" OR "experiential knowledge" OR "authentic experience" OR interview* OR opinion OR qualitative OR "mixed methods" OR "action research" OR "feminist research" OR "ground theory" OR phenomenology ) AND TITLE-ABS-KEY ( psychological OR "psychological health" OR "psychological well-being" OR "Mental health" OR "mental health disorder" OR "mental health challenges" OR "mental well-being" OR "mental illness" OR "psychiatric disorder" OR "mood disorder" OR "eating disorders" OR "sleep disorders" OR anxiety OR depression OR " Emotional disorders" OR "emotional challenges" OR "emotional well-being" OR "emotional problems" OR "emotional distress" OR "Psychosocial disorders" OR "psychosocial challenges" OR "psychosocial well-being" OR "psychosexual difficulties" OR "social isolation" OR "social anxiety" OR "self-esteem" OR "self-image" OR "body image" OR "sexual functioning" OR "sexual disorder" ) ) AND ( EXCLUDE ( DOCTYPE , "no" ) OR EXCLUDE ( DOCTYPE , "sh" ) OR EXCLUDE ( DOCTYPE , "le" ) OR EXCLUDE ( DOCTYPE , "ch" ) ) AND ( LIMIT-TO ( LANGUAGE , "English" ) ) |
| --- |

Results = 162

**PubMed**

| 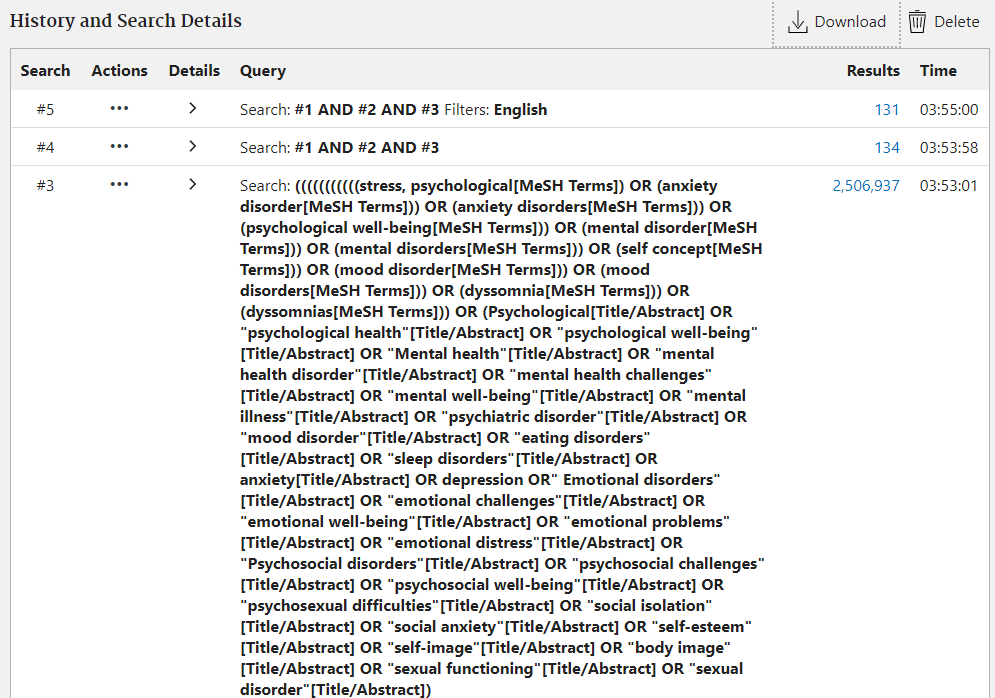  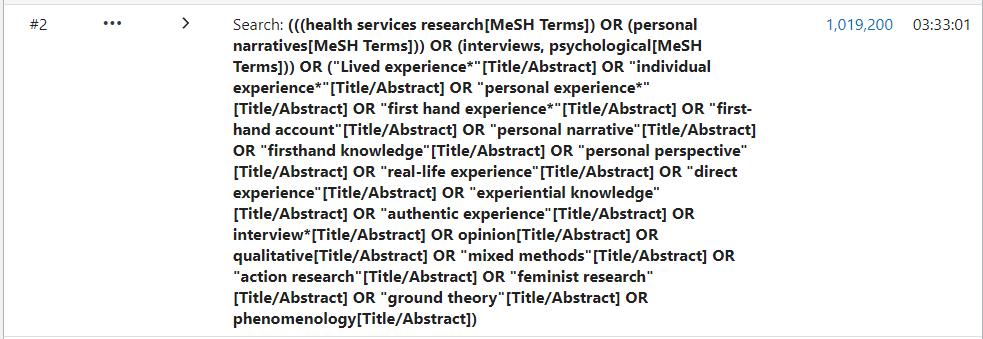  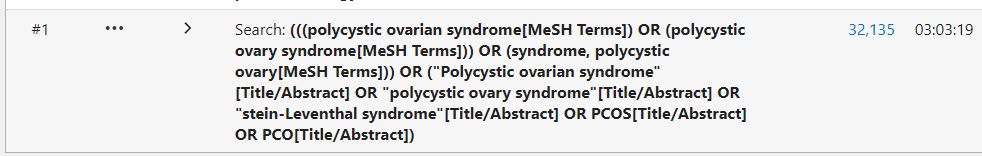 |
| --- |

Results = 131

**Cochrane Reviews and Trials**

| 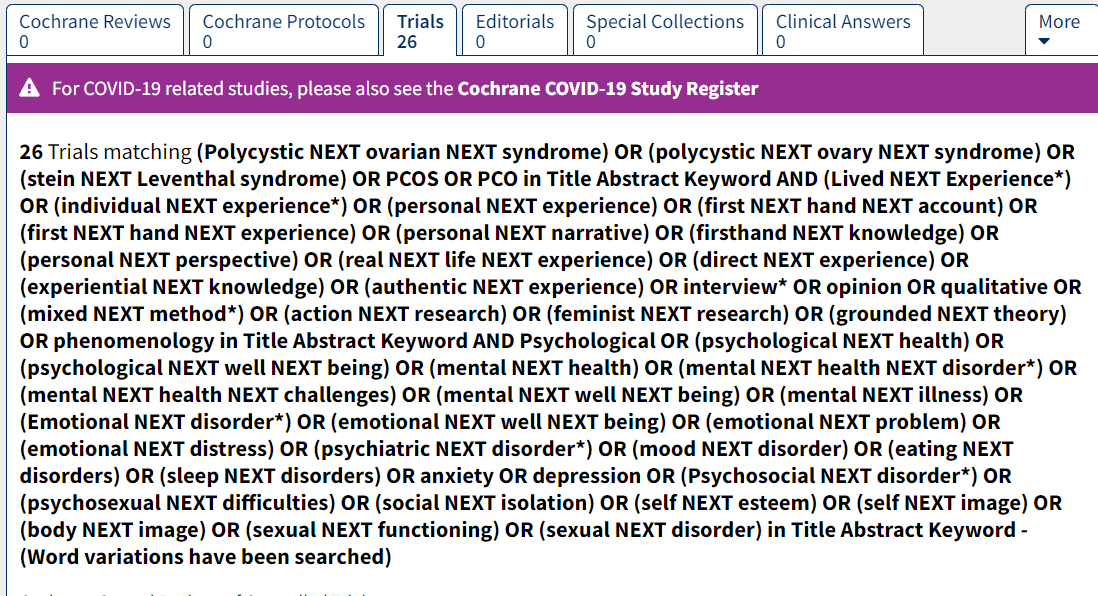 |
| --- |

Results = 26

**MEDLINE**

| Pdf |
| --- |

Results= 107

**CINAHL**

| Pdf |
| --- |

Results= 48

**Duplicate references** = 313 (in Trash)

## Supplementary Section 3 (S3): Illustrative Quotes

**Theme 1: Mental Health Challenges**

Subtheme: Depression

*"Invisible on the outside yet still visible in the eyes at times. Mental pain. Physical pain. Existential pain." (Canada^51^)(p.03)*

*“In this disease, you are always depressed and in a bad mood.” (Iran^4^)(p.394)*

Subtheme: Hopelessness

*“I am not interested in daily tasks.” (Iran^45^)(p.04)*

Subtheme: Anxiety, Fear of infertility and childlessness

*“…I'm always stressed. In terms of how I manage it. I tend to resort to quick fixes.” (Australia^57^) (p.2048)*

Subtheme: Anxiety, Uncertainty and Long-Term Health Anxiety

*"I just don’t know when I will have menstruation or when I will be pregnant. This is the uncertainty" (China*^58^*) (p.300)*

Subtheme: Body Image Dissatisfaction, Unattractiveness & Low Self-Worth

*"I am anxious about my body image, as my dress style is now limited. I no longer wear skirts or shorts, instead opting for loose pants or long skirts. This makes me feel inferior to others." (China^52^) (p.04)*

Subtheme: Body Image Dissatisfaction, Feeling “Less Feminine”

*"The fertility problem is more important to me than the excess hair growth or obesity. I can solve the excessive hair and obesity, but not the fertility problem. I feel that if I can't bear a child; I will lose all sense of being a woman.” (Iran^69^) (p.05)*

**Theme 2: Psychosocial Challenges**

Subtheme: Social comparison

*“…I got depressed when I compare myself with them; then, I prefer to stay at home.” (Iran^69^) (p.05)*

Subtheme: Societal judgement and stigma

*“I find that the pressures imposed by society on women to be thin and have beautiful hair are in direct opposition with the symptoms I have experienced as a result of PCOS.” (UK^66^) (p.05)*

*“…she said, ‘a single girl shouldn’t have such a big belly’, and I got so embarrassed.” (Iran^69^)(p.05)*

*“Yeah, you don’t want to bum everyone out.” (USA^62^)(p.07)*

Subtheme: Social Isolation

*“I try not to be in touch with my friends. I’ve only told my best friend about my polycystic ovary syndrome.” (Iran^44^)(p718)*

*“Often exercise I don't want to do it outside of the house at all. I don't want people to see me even walking down the street.” (Australia^48^)(p.05)*

Subtheme: Intimacy and Relationship Strain

*“I have always felt that I should be grateful that any man wants to be with me – with my spots and hairs and obesity.” (Canada^51^) (p.04)*

Subtheme: Sociocultural Variation in Psychosocial Challenges

*"Given that the festival and wedding season lasts for approximately half the year… this can be a socially tricky situation to navigate" (India^71^)(p334)*

## Supplementary Section 4 (S4): Additional Synthesized Findings

Synthesized Finding 3: Psychological impact of PCOS related to diagnosis

Women with PCOS frequently faced delayed diagnoses, often consulting multiple practitioners before receiving a diagnosis, which lead to frustration and distress^60, 66^. Women expressed feelings of dismissal by their healthcare providers, often receiving inadequate explanations that heightened anxiety about their long-term health ^63, 75^. Noteworthy, some women had to self-advocate for referrals, as their concerns were often overlooked ^76^. This is reiterated by women who found that doctors narrowly focussed on weight management, which left many feeling unheard and inadequately treated ^60, 63, 75^.

Synthesized Finding 4: Psychological impact of PCOS related to management

Women often faced a one-size-fits-all approach to management, with doctors primarily advising weight loss, which led to frustration and anger ^60, 75^. Women felt abandoned by their doctors post-diagnosis due to the lack of individualized, structured management plans, leaving them uncertain about how to proceed ^77^. Furthermore, the lack of effective treatment options and poor follow-up care contributed to feelings of hopelessness, causing some to forgo medical support altogether^77^.

Synthesized Finding 5: Coping with PCOS

The data demonstrate that women with PCOS find relief through shared experiences via online support forums and peer groups, which reduced isolation, and addressed gaps in medical guidance^60, 78^. Additionally, self-education using PCOS resources enabled women to take control of their health, improving self-advocacy and confidence in navigating medical consultations^79^. Cognitive coping mechanisms, such as acceptance of the condition, played a role in developing emotional resilience^26^. Furthermore, women expressed a need for greater support from peers and family^54^, as well as compassionate and proactive healthcare providers^75^.

## Supplementary Information Section 5 (S5): **ConQual summary of findings**

**Table 4:** ConQual summary of findings

| **Systematic review title: The Lived Experiences of Women with Polycystic Ovary Syndrome and its Psychological Challenges: A Systematic Review and Meta-Analysis** | | | | | |
| --- | --- | --- | --- | --- | --- |
| **Population:** Women diagnosed with PCOS based on an established criteria such as the Rotterdam | | | | | |
| **Phenomena of interest:**The psychological impact of PCOS on women, including mental health, emotional well-being, and psychosocial experiences. | | | | | |
| **Context:** This qualitative SR synthesizes findings from studies conducted across diverse settings, including healthcare facilities, community-based focus groups, telephonic interviews, and online forums. | | | | | |
| **Synthesised findings** | **Type of research** | **Dependability**^1^ | **Credibility**^2^ | **ConQual^3^score** | **Comments** |
| **Mental Health Challenges of PCOS** include depression, anxiety, and body image dissatisfaction. The unpredictability of symptoms contributes to a perceived loss of control, while fear of infertility and long-term health uncertainty exacerbate distress. Body image concerns, particularly unattractiveness and feeling "less feminine," negatively impact self-worth and emotional well-being. | Qualitative | Downgrade 1 level* | High (remains unchanged) | Moderate | Multiple participant quotes across studies present similar findings.  Out of 98 findings extracted from 44 studies, the majority of findings (95) were unequivocal and 3 were credible.  Although Jones et al. (2024) provided credible findings, the supporting quotes were fragmented, referencing specific words or phrases from multiple participants rather than complete, standalone statements. |
| **Psychosocial Burden of PCOS** PCOS significantly affects social relationships, leading to social comparison, judgment, stigma, and isolation. Visible symptoms, such as weight gain, acne, and hirsutism, result in public scrutiny and emotional distress. Women face challenges in intimate relationships, with infertility and body image concerns contributing to emotional disconnection, reduced libido, and relationship strain. | Qualitative | Downgrade 1 level* | Downgrade 3 levels | Moderate | Many quotes throughout studies support findings.  Out of 40 findings extracted from 18 studies, the majority of findings (37) were unequivocal, 3 findings were credible, 1 finding was unsupported.  Despite a few credible and one unsupported finding, the majority of findings were unequivocal, warranting a moderate ConQual rating for this synthesized finding. |
| **Sociocultural variation** shapes the psychosocial impact of PCOS, influencing beauty standards, infertility distress, and workplace stigma. | Qualitative | Downgrade 1 level* | Downgrade 2 levels | Low | Limited quotes supported the findings.  Out of 7 findings extracted from 7 studies, 6 were unequivocal, 1 was credible |

^1Is there congruity between the research methodology and the research question or objectives?^

^Is there congruity between the research methodology and the methods used to collect data?^

^Is there congruity between the research methodology and the representation and analysis of data? Is there a statement locating the researcher culturally or theoretically?^

^Is the influence of the researcher on the research, and vice-versa, addressed?^

^2Unequivocal (findings accompanied by an illustration that is beyond reasonable doubt and; therefore not open to challenge, 0 or -1 level).^

^Credible (findings accompanied by an illustration lacking clear association with it and therefore open to challenge, -2 levels).^

^Unsupported (findings are not supported by the data, -3 levels)^

^3High, Moderate, Low, Very Low^

^*Downgraded one level due to prevalent dependability concerns across the included primary studies. While 25 studies provided a statement locating the researcher culturally or theoretically, 18 did not, and only 6 acknowledged the researcher's influence on the study.^
